# Huge Benign Ovarian Cystic Teratoma in a Patient with a History of Hansen's Disease

**DOI:** 10.1155/2014/345767

**Published:** 2014-08-26

**Authors:** Patrick I. Okonta, Chukwuemeke Mofon

**Affiliations:** Department of Obstetrics and Gynaecology, Delta State University Teaching Hospital, Oghara 331101, Delta State, Nigeria

## Abstract

Mature ovarian cystic teratomas are common benign ovarian neoplasm derived from germ cells. With increasing availability of ultrasound services even in developing countries, the diagnosis of benign ovarian tumour is made earlier and the size of the ovarian tumour at diagnosis is relatively small. It is unusual to find an ovarian cystic teratoma larger than 10 cm. We report a huge mature ovarian cystic teratoma in a multipara with a history of Hansen's disease. We conclude that, in circumstances where women have restricted access to health care, the unusual finding of mature ovarian cystic teratoma larger than 10 cm is possible due to delayed presentation for diagnosis and treatment.

## 1. Introduction

Mature ovarian cystic teratomas are common benign ovarian neoplasm derived from germ cells. Histologically, they are composed of variable proportions of tissue originating from the ectoderm, mesoderm, and endoderm [1]. Cystic teratomas are commonly seen in active reproductive years but can occur in any age group and may be seen in postmenopausal women [2].

With increasing availability of ultrasound services even in developing countries, the diagnosis of benign ovarian tumour is made earlier and the size of the ovarian tumour at diagnosis is relatively small. It is unusual to find an ovarian cystic teratoma larger than 10 cm [3, 4]. We report a huge mature ovarian cystic teratoma in a multipara with a history of Hansen's disease.

## 2. Case Report

**Figure 1:**
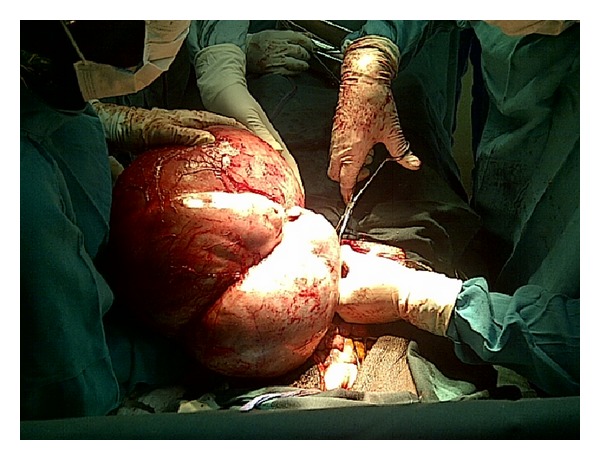
The huge mature ovarian cystic teratoma before excision.

**Figure 2:**
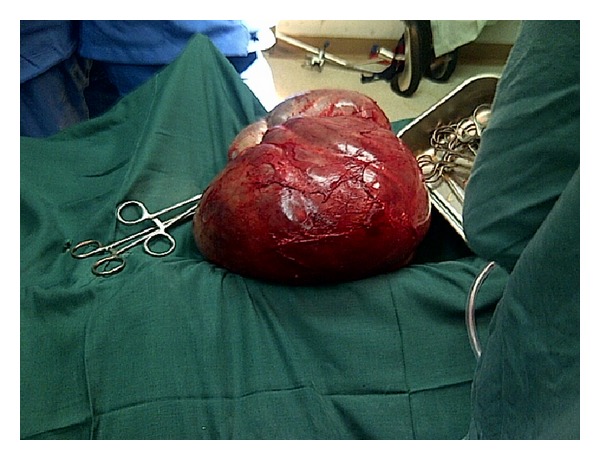
The ovarian tumour excised.

**Figure 3:**
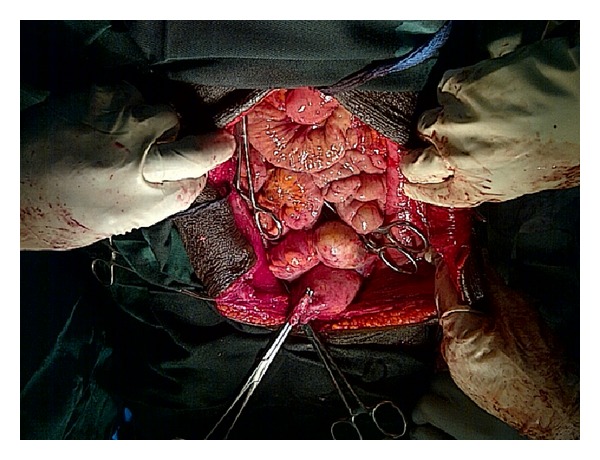
The abdominal and pelvic cavity after excision of the mature ovarian cystic teratoma. Note the normal uterus.

Mrs. O.B. is a 31-year-old Para 6 (6 alive) woman who presented with a 2-year history of abdominal swelling. Swelling was localized to the lower abdomen, was initially small, and had been progressively increasing in size. There was associated dull continuous abdominal pain localized to the lower abdomen. There was no associated vomiting or change in bowel habits and no urinary symptoms or change in menstrual pattern. She was diagnosed with leprosy in 1995 and had been treated. She had been in remission since 1999. Physical examination revealed a small statured woman with hypopigmented lesions on both extremities and missing proximal phalanges. She was not pale, febrile, or icteric. Her respiratory rate was 16 cycles per minute and the chest was clinically clear. Her pulse rate was 88 beats per minute and the blood pressure was 106/70 mmHg. The abdomen was distended, soft with no area of tenderness. There was a soft, cystic abdominal mass which was not distinctly palpable per abdomen. Pelvic examination revealed a normal vulva and vagina. The cervix was healthy looking, and the os was closed. The uterus was anteverted and not bulky. An abdominal ultrasound done revealed a large multiseptated cystic mass in the abdomen and pelvis measuring in part 25.2 × 20.9 × 17.5 cm and containing low level echogenic debris. The uterus was normal. Her haemoglobin was 9.6 g/dL, white cell count was 6,300/*μ*L, and urinalysis was normal. A diagnosis of an ovarian tumour was made and she was prepared for exploratory laparotomy. She was given haematinics and micronutrients and was advised to have adequate nutrition in order to increase her haemoglobin concentration as well as her general nutritional state. Two pints of blood was crossmatched and made available for the surgery. She had an exploratory laparotomy and the finding was a large multiloculated right ovarian cyst measuring 28 × 25 × 19 cm and weighing 9 kg (Figures 1 and 2). The left ovary was grossly not affected and the uterus was normal in size (see Figure 3). Histopathological examination of the excised cyst showed that it was a benign cystic teratoma. Her postoperative recovery was uneventful and she was discharged on the 6th postoperative day. She was seen regularly on follow-up at the outpatient gynaecology clinic and sonographic assessment of the remaining left ovary was done to rule out any development of a cyst.

## 3. Discussion

Mature cystic teratoma also known as benign cystic teratoma or dermoid cyst comprises approximately 10–20% of all ovarian neoplasms and 60% of all benign neoplasms [5]. These tumors are typically slow growing and most measure between 5 and 10 cm and are bilateral in approximately 10% of cases [4, 6]. Microscopically, endodermal or mesodermal derivatives may be found but ectodermal elements predominate. They may contain skin, hair follicle, and sweat gland and occasionally contain sebum, blood, bones, nail, and teeth. Dermoid cysts usually present with abdominal swelling, discomfort, and pain [7, 8]. The case of huge mature cystic ovarian teratoma presented above had been diagnosed with Hansen's disease almost 20 years prior to presentation to us. She had been treated and declared free from the disease subsequently. Hansen's disease is an infectious chronic granulomatous disease caused by the bacillus* Mycobacterium leprae*. It primarily affects the peripheral nerves, skin, upper respiratory tract, eyes, and nasal mucosa. To the best of our knowledge, this is the only reported case on mature cystic ovarian teratoma in a patient with a previous history of Hansen's disease. There is no reason to ascribe a causal association between Hansen's disease and the development of the teratoma. However, the fact that the patient had suffered from Hansen's disease and is living in an isolated community (leprosarium) could have hindered her early access to health care. Her consultation and surgery were sponsored by a nongovernmental organization engaged in works of charity. This highlights the difficulties, burden, and challenges persons with a past history of such stigmatized disease as Hansen's face even after cure [9, 10].

A significant point of this case is the fact that though the tumour was large and had probably developed over a long period, there was no evidence of malignant transformation. Tumour size has been documented as a risk factor for malignant transformation [5, 11]. However, malignant transformation of benign cystic teratoma is rare, occurring in 2% of cases usually in older women and when it occurs, squamous cell carcinoma is the most common [2, 3, 12]. Other complications of benign cystic teratoma include rupture into the peritoneal cavity or an adjoining viscera, torsion, infection, and haemorrhage [4, 13].

It is important during surgery to carefully inspect the contralateral ovary for any involvement. Previously, a biopsy of the grossly normal contralateral ovary was made to rule out any possible involvement. However, this practice is no longer recommended; instead serial follow-up scan is used to monitor the ovary [14, 15].

## 4. Conclusion

In circumstances where women have restricted access to health care, the unusual finding of mature ovarian cystic teratoma larger than 10 cm is possible due to delayed presentation for diagnosis and treatment.
